# Promyelocytic leukemia zinc-finger induction signs mesenchymal stem cell commitment: identification of a key marker for stemness maintenance?

**DOI:** 10.1186/scrt416

**Published:** 2014-02-24

**Authors:** Farida Djouad, Gautier Tejedor, Karine Toupet, Marie Maumus, Claire Bony, Anne Blangy, Paul Chuchana, Christian Jorgensen, Danièle Noël

**Affiliations:** 1 Inserm, U844, Hôpital Saint Eloi, Montpellier F-34091, France; 2 Université MONTPELLIER1, UFR de Médecine, Montpellier F-34000, France; 3 Service d’Immuno-Rhumatologie Clinique, Hôpital Lapeyronie, Montpellier F-34295, France; 4 Centre de Recherche de Biochimie Macromoléculaire, CNRS-UMR 5237–1919, Montpellier F-34293, France; 5 Université MONTPELLIER2, Montpellier F-34000, France

## Abstract

**Introduction:**

Mesenchymal stem cells (MSCs) are an attractive cell source for cartilage and bone tissue engineering given their ability to differentiate into chondrocytes and osteoblasts. However, the common origin of these two specialized cell types raised the question about the identification of regulatory pathways determining the differentiation fate of MSCs into chondrocyte or osteoblast.

**Methods:**

Chondrogenesis, osteoblastogenesis, and adipogenesis of human and mouse MSC were induced by using specific inductive culture conditions. Expression of promyelocytic leukemia zinc-finger (PLZF) or differentiation markers in MSCs was determined by RT-qPCR. PLZF-expressing MSC were implanted in a mouse osteochondral defect model and the neotissue was analyzed by routine histology and microcomputed tomography.

**Results:**

We found out that PLZF is not expressed in MSCs and its expression at early stages of MSC differentiation is the mark of their commitment toward the three main lineages. PLZF acts as an upstream regulator of both Sox9 and Runx2, and its overexpression in MSC enhances chondrogenesis and osteogenesis while it inhibits adipogenesis. *In vivo*, implantation of PLZF-expressing MSC in mice with full-thickness osteochondral defects resulted in the formation of a reparative tissue resembling cartilage and bone.

**Conclusions:**

Our findings demonstrate that absence of PLZF is required for stemness maintenance and its expression is an early event at the onset of MSC commitment during the differentiation processes of the three main lineages.

## Introduction

Bone marrow stromal cells, commonly referred to as mesenchymal stem cells (MSCs), are nonhematopoietic cells that display differentiation capacity toward adipocytes, osteoblasts, chondrocytes. MSCs exist in almost all tissues and are a key cell source for tissue repair and regeneration [1]. Stem-cell maintenance in the adult organism is essential for tissue homeostasis initiation of native tissue regeneration and the response to injury. To maintain the stem cell pool in the niche, some stem cells must remain undifferentiated and quiescent. Under specific circumstances, they can proliferate through cyclic mitotic divisions. Cells committed to differentiate enter the meiotic pathway, which comprises a unique program of gene expression and chromatin remodeling. How does each MSC decide whether to proliferate or differentiate? Although the molecular mechanisms controlling this delicate balance are largely unknown, critical transcription factors involved in the commitment of different MSC-derived lineages have been identified [2,3]. For example, it is well established that Sox9 is the master factor that regulates chondrogenesis, whereas osteoblastic differentiation is controlled by Runx2, and PPAR-γ is involved in adipocyte commitment [4]. The possibility that an intrinsic molecule acting upstream these latter transcription factors can subsequently control MSC “stemness” properties or lineage-specific commitment has not been described. If such a factor does exist, it would help to understand the biology of MSC and the mechanisms regulating their differentiation.

PLZF (promyelocytic leukemia zinc-finger) also known as, ZBTB16, ZNF145 or Kruppel-like zinc-finger protein, belongs to the family of the transcriptional repressors POK (POZ and Krüppel). In addition to nine Krüppel-type sequence-specific zinc fingers, PLZF contains a conserved POZ (poxvirus and zinc finger) domain in its N terminus [5]. This domain mediates protein-protein interactions and allows POZ domain-containing proteins to be involved in several differentiation pathways, including osteoclastogenesis, hematopoiesis, adipogenesis, and muscle differentiation [6]. PLZF expression profile in early, but not in differentiated hematopoietic cells, suggests its involvement in stem cell maintenance and self-renewal [7]. Moreover, PLZF-knockout mice exhibit defects in patterning of the limb and axial skeleton [8]. Together with Gli3 (GLI-Kruppel family member 3), PLZF has been described to be specifically required for stylopod and zeugopod element formation at very early stages of limb development, before the initiation of cartilage condensations [9]. In line with these *in vivo* studies, it has been shown that PLZF overexpression in human MSCs enhances chondrogenesis [10]. In addition, PLZF was described to play an important role in early osteoblastic differentiation [11,12]. Regarding adipogenesis, its overexpression was reported to be repressive, and conversely, RNAi-mediated knockdown of PLZF enhances adipogenesis [13]. This is in contrast with results described by Liu and co-workers [10], showing that PLZF knockdown in MSC decreases adipogenic genes as well as lipid deposit during adipogenesis. All together, these studies suggest that PLZF acts as a key factor in MSC differentiation processes. However, because it is well established that a competition or mutual suppression exists between the genetic pathways that lead to either chondrocyte or osteoblast differentiation in mesenchymal progenitors [14], it is still not clear whether PLZF acts as an inducer of chondrogenesis or osteogenesis.

In early mesenchymal condensations, MSC possess multidifferentiation potential as they coexpress Sox9, Runx2, and PPAR-γ [15]. This suggests that these transcription factors are regulated by other factors to initiate MSC differentiation toward chondrogenic or osteogenic programs. In the present study, we investigated whether PLZF may act in concert with these transcription factors and play a role in the commitment and differentiation of MSC toward multiple mesodermal lineages.

## Materials and methods

### Isolation of MSC

The mesenchymal progenitor cell line C3H10T1/2 (thereafter called C3) [16] was grown in complete Dulbecco modified Eagle medium (DMEM; Sigma, l’Isle d’Abeau, France) supplemented with 10% fetal calf serum (FCS) (Hyclone, Perbio, Bezons, France), 2 m*M* glutamine, 100 U/ml penicillin, and 100 μg/ml streptomycin (Invitrogen, Cergy, France). Primary MSC were isolated from C57BL/6 (mMSC). Bone marrow was flushed out of long bones, and the cell suspension (0.5 × 10^6^cells/cm^2^) was plated in minimum essential medium (MEM)-α supplemented with 10% fetal bovine serum (FBS) (Hyclone, Thermo Fisher Scientific, Brebières, France), 2 m*M* glutamine, 100 U/ml penicillin, 100 mg/ml streptomycin (Lonza, Levallois-Perret, France), and 2 ng/ml human basic fibroblast growth factor (bFGF) (R&D Systems, Lille, France). At subconfluence, cells were replated at the density of 5,000 cells/cm^2^ and used before passage 10. Human MSC (hMSC) were isolated from patients after written informed consent and approval by the General Direction for Research and Innovation, the Ethics Committee from the French Ministry of Higher Education and Research (registration number: DC-2009-1052). Bone marrow from trabeculae of bone specimens was flushed and expanded in minimum essential medium α (MEMα; Invitrogen) supplemented with 10% FCS, 2 m*M* glutamine, 100 U/ml penicillin, 100 μg/ml streptomycin, and 1 ng/mlL basic fibroblast growth factor (bFGF; R&D Systems, Lille, France). Confluent hMSCs were used between passage 2 and 4 to ensure homogeneous cell populations. Before use, hMSC populations were phenotyped by flow cytometry (cells were negative for CD34 and CD45 and positive for CD90, CD105, and CD73).

### Plasmid and transfections

pSG5 Expression vector (Stratagene, La Jolla, CA, USA) containing PLZF (pSG5-PLZF) was a gift from J.D. Licht and P.J. Martin [17]. C3 cells were stably transfected by using the calcium-phosphate precipitation technique with the pSG5-PLZF and pX343 plasmid carrying the hygromycin B resistance gene as a selectable marker. Clones were isolated from individual colonies and amplified before analysis with RT-qPCR and immunofluorescence by using anti-PLZF antibody (Santa Cruz Biotechnology, Le Perray en Yveline, France).

### Cell differentiation

Differentiation of MSC was induced by culture in specific conditions for 21 days. For adipogenesis, murine MSCs were plated at 10^4^ cells/cm^2^ in complete Dulbecco modified Eagle medium (DMEM)-F12 (Invitrogen) with 16 μ*M* biotin, 18 μ*M* panthotenic acid, 100 μ*M* ascorbic acid, 5 μg/ml insulin, 0.03 μ*M* dexamethasone, 1 μg/ml transferrin and 2 ng/ml triiodothyronine (T3) (Sigma-Aldrich). Human MSCs were plated at the density of 8 × 10^3^ cells/cm^2^ in DMEM-F12 containing 5% newborn calf serum, 1 μ*M* dexamethasone, 50 μ*M* isobutyl-methylxanthine, and 60 μ*M* indomethacin. The formation of lipid droplets was visualized with Oil Red O staining. For osteogenesis and chondrogenesis, inductive conditions specific for murine or human MSCs were as already reported [18,19]. After osteogenic differentiation, mineralized extracellular matrices were visualized after fixation in 95% ethanol for 30 minutes and staining with a 2% Alizarin Red S solution, pH 4.2 (Sigma) [20]. Chondrogenesis was assessed by immunohistochemistry on paraffin sections of pellets by using an anti-collagen II antibody (Interchim, Montluçon, France) and the Ultravision Detection System Anti-polyvalent HRP/DAB kit (Lab Vision, Francheville, France).

### Microarray analysis

Hybridization of Affymetrix HG-U133 plus 2.0 arrays was described in a previous study [19]. Data can be found under the GEO accession number GSE10315 [21]. Raw gene-expression data were processed for normalization and signal calculation with the Expression Variation software previously described [22]. To determine differentially expressed genes, comparative occurrence analysis was performed by using a recently described approach [23].

### RNA extraction and quantitative PCR

Total RNA was isolated by using RNeasy mini kit (Qiagen S.A., Courtaboeuf, France) and reverse-transcribed by using GeneAmp Gold RNA PCR Core kit (Applied Biosystems). Reverse transcriptase quantitative PCR (RT-qPCR) was performed by using LightCycler 480 SYBR Green I Master mix and real-time PCR instrument (Roche Applied Science, Meylan, France). Primers were designed by using the web-based applications, Primer3 and BLAST at the National Center for Biotechnology Information. Expression of the housekeeping gene encoding ribosomal protein S9 (RPS9) was measured for normalization. The relative amount of a given mRNA was calculated by using the formulae 2^-∆Ct^ or 2^-∆CCt^.

### Coloration CM-DiI

Stock solution of the fluorescent cell-tracer CM-DiI (Molecular Probes, Interchim, Montluçon, France) was reconstituted at a concentration of 1 μg/μl in dimethyl sulfoxide (DMSO) and used to label C3 and PLZF-expressing C3 cells (C3-PLZF), as described previously [24].

### Tibial implantation of C3 and C3-PLZF MSCs

The 21- to 23-week-old female severe combined immunodeficient (SCID/Beige) or DBA/1 mice (five per group) were grown in our animal facilities. The protocol was approved by the Committee on the Ethics of Animal Experiments in Languedoc-Roussillon (CEEA-LR 36) (Permit Number: CEEA-LR-1043). Intratibial injection model was used to create cartilage and bone lesions and to inject either C3 or C3-PLZF cells (2.5 × 10^5^ cells) suspended in 10 μl of PBS in the right tibia, as previously described [25]. In brief, the mice were anesthetized by using 1.5% to 2% isoflurane and oxygen in induction chamber. A 3-mm longitudinal incision was made over the patellar ligament with a scissor. A 25-gauge needle was introduced in the intraarticular space and inserted through the proximal tibial plateau to inject the C3 or C3-PLZF cells into the medullary cavity. The overlying skin incision was sutured, and animals were allowed immediate postoperative weight-bearing. On day 28 after cell injection, mice were killed, and the injected tibiae were submitted to micro-computed tomography (μCt) and histologic analysis.

### Micro-computed tomography

After fixation in 4% formaldehyde for 1 week, entire tibiae were scanned with a micro-computed tomograph (SkyScan 1076; Kontich, Belgium). Each scan was performed by using a 0.025-mm titanium filter and a pixel size of 9 μm. This provides image data of the mineralized, subchondral bone in both tibia and femur. Then a 3D image was reconstructed by using NRecon software (SkyScan NRecon version 1.6.6). Misalignment compensation, ring artifacts, and beam-hardening were adjusted to obtain a correct reconstruction of the joint and long bones. Bone mineral density (BMD) was evaluated with CT Analyser software (SkyScan CT Analyser version 1.8.1.4). Calibration was made on μCT images performed on a tube of water and two BMD rods with BMD values of 0.25 and 0.75 g/cc, respectively. These three scans were achieved at the same time and by using the same parameters as for the tibias. After calibration, BMD was calculated for each knee joint in a well-defined region of interest corresponding to the area where PBS or cells were injected (from the beginning of the tibiae to 2.321 mm of depth).

### Histology and immunohistochemistry

Tibias were fixed in 4% paraformaldehyde during 1 week, decalcified in 5% formic acid for 3 days, and processed for routine histology. Paraffin-embedded tissue sections (5 μm) were rehydrated through a gradient of toluene and alcohol and either stained with safranin O and fast green before examination by light microscopy or mounted in fluorescent mounting medium (Dako, Trappes, France) for red fluorescence visualization. Paraffin sections were also stained with Goldner trichrome, as described previously [26] to visualize bone in green and bone marrow in dark purple.

For immunohistochemistry, we used the Ultravision detection system anti-polyvalent HRP/DAB kit (Lab Vision; Microm, Francheville, France), according to the manufacturer’s instructions. For type II collagen immunostaining, samples were first incubated with pepsin (Sigma) for epitope retrieval. Primary antibody anti-type II collagen monoclonal mouse antibody (1:50; Interchim) was incubated for 2 hours at RT. Samples were finally counterstained with Mayer hematoxylin (Lab Vision) for 3 minutes and mounted with Eukitt (Sigma). Immunostaining was imaged with the Digital slide scanner NanoZoomer 2.0-HT (Hamamatsu Ltd, Japan). Immunopositive extracellular matrix showed a brown staining.

### Statistical analysis

Data are expressed as the mean ± SEM of at least three independent experiments. Student *t* test was used to compare two treatment groups, and multiple comparisons were performed by ANOVA corrected by Bonferroni posttest (***P < 0.001; **P < 0.01; and *P < 0.05).

## Results

### Induction of PLZF expression during chondrogenesis

**Figure 1:**
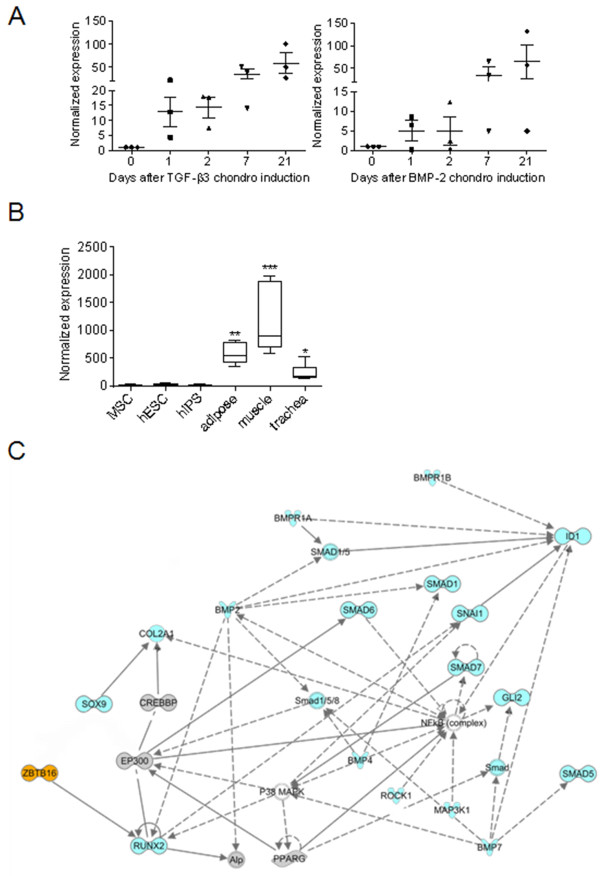
**Absence of PLZF expression in cells signs their stemness state. (A)** Expression profile of PLZF during the chondrogenic differentiation of MSCs as assessed by microarray analysis. At day 0, absence of PLZF expression was defined as 1, and this was used to calculate the relative expression at the remaining time points. **(B)** PLZF expression profile in human MSCs, ESCs, iPSs, adipose tissue, skeletal muscle, and trachea as reported in the Amazonia website. The signal intensity is shown on the y axis as arbitrary units determined by the GCOS 1.2 software (Affymetrix). When indicated, the mean expression is significantly different from that in MSCs, hESCs, and hiPS. **(C)** PLZF-related network was reconstructed by using the Ingenuity Pathways Analysis Software (IPA) software where PLZF acts upstream of Runx2 and regulates downstream genes. Blue-shaded genes are genes identified to be involved in chondrogenesis, grey-shaded genes either involved in osteogenesis or adipogenesis, and unshaded genes are those associated with the identified genes based on pathway analysis.

In a previous study, we performed a transcriptomic analysis of MSCs isolated from three different donors and differentiated into chondrocytes by using the micropellet culture conditions and either TGF-β3 or BMP-2 as inducers, or osteoblasts or adipocytes. Their mRNA profiles were analyzed at different time points by using Affymetrix gene chips [19]. *In silico* analysis was performed by varying the stringency for selection of regulated genes. Parameters including normalized fold change (FC) over control value at day 0 (D0), occurrence in the samples depending on the culture conditions, the donor, or the time point, were adjusted. We found that PLZF ranked first of 1,248 transcription factors (TFs) at 7 and 21 days of chondrogenesis, whatever the chondrogenic inducer (data not shown). At earlier time points, PLZF expression was greatly induced by TGF-β3, ranking third and fifth of the TF at days 1 and 3, respectively. An early induced PLZF expression was also observed from day 3 to day 21 of osteogenesis and at days 1 and 3 of adipogenesis with an FC >2.5 in both cases remaining stable at all indicated time points (data not shown). Interestingly, PLZF was not expressed in any of the three MSC samples (at day 0). By RT-qPCR, we confirmed that its relative expression during either TGF-β3 or BMP-2-dependent *in vitro* chondrogenesis was substantially induced (by a 15-fold or 5-fold factor, respectively) as soon as day 1 of the differentiation process (Figure 1A). To address whether the absence of PLZF expression was observed in other MSC samples or stem cells from different origins, we assessed PLZF expression in pluripotent cells including human embryonic stem cells (hESC) and induced pluripotent stem cells (hiPS). We also analyzed different mesenchymal tissues such as adipose tissue, skeletal muscle, and trachea, by using the Amazonia website [27], which is dedicated to relative comparison of data collected from analysis of Affymetrix U133 Plus 2.0 microarrays. In line with our results, the absence of PLZF expression was confirmed in undifferentiated MSCs (4 more samples), and also in hESC (25 samples) and hiPS (34 samples). Moreover, we observed a substantial expression of PLZF in adipose tissue, skeletal muscle and trachea compared with the stem cells (Figure 1B). We then constructed a regulatory module to infer the underlying relations between PLZF and genes involved in the sequence of events of chondrogenesis [28], genes related to adipogenesis or osteogenesis. A relation between PLZF and predicted target-gene groups was observed. Indeed, by using the Ingenuity software to analyze the PLZF-related predicted network, we found that PLZF might act upstream of the master regulator of osteogenesis Runx2 and also regulate at least 19 genes related to the chondrogenesis process and PPARγ, the key regulator of adipogenesis (Figure 1C). These results suggest that absence of PLZF expression might be a signature of the undifferentiated state of multipotent stem cells, whereas its expression appears in committed cells.

### PLZF expression signs the commitment of MSCs

**Figure 2:**
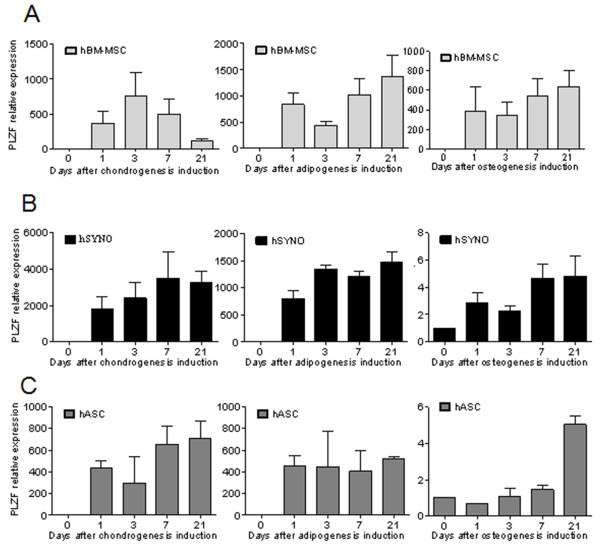
**PLZF expression profile in the course of MSC differentiation into the three main lineages.** Expression profile of PLZF during the differentiation of human **(A)** bone marrow- (hBM-MSC), **(B)** synovium- (hSYNO), and **(C)** adipose-derived (hASC) MSCs toward the chondroblastic, osteoblastic, and adipocytic lineages by using RT-qPCR. RT-qPCR data represent the mean ± SEM of three independent experiments.

To define whether the induction of PLZF expression in MSCs is restricted to the chondrogenic program, we investigated its expression profile in MSCs undergoing osteogenic or adipogenic differentiation. We therefore assessed the expression profile of PLZF during the differentiation process of bone marrow-derived MSCs (BM-MSCs) toward the chondroblastic, osteoblastic and adipocytic lineages by using real-time RT-PCR. We observed an upregulation of PLZF expression level as soon as day 1 of MSC differentiation toward the three lineages. The increased expression level of PLZF was noticeable until the end of the differentiation processes, compared with undifferentiated MSCs (Figure 2A). However, PLZF expression was stable from day 1 to day 21 during adipogenesis and osteogenesis, whereas it peaked at day 3 and decreased thereafter during chondrogenesis. We next investigated the expression profile of PLZF during the differentiation of synovium- (hSYNO) and adipose tissue-derived MSC (hASC) that we previously described for sharing similar differentiation capacities with BM-MSC [29,30]. We showed that, whatever was the MSC tested, PLZF was induced as early as day 1 of chondrogenesis, adipogenesis, and osteogenesis and maintained during the differentiation (Figure 2B, C). With the exception of hASC, the expression of PLZF remained stable or slightly increased during adipogenesis or osteogenesis of both MSC sources. Similar behavior was observed for chondrogenesis of hSYNO and hASC. Although some variations of PLZF expression was observed, depending on the lineage induced and the origin of MSC, its high expression at day 1 of MSC differentiation was indicative of commitment of MSCs toward the three main lineages.

### PLZF regulates the osteogenic and chondrogenic master regulators

**Figure 3:**
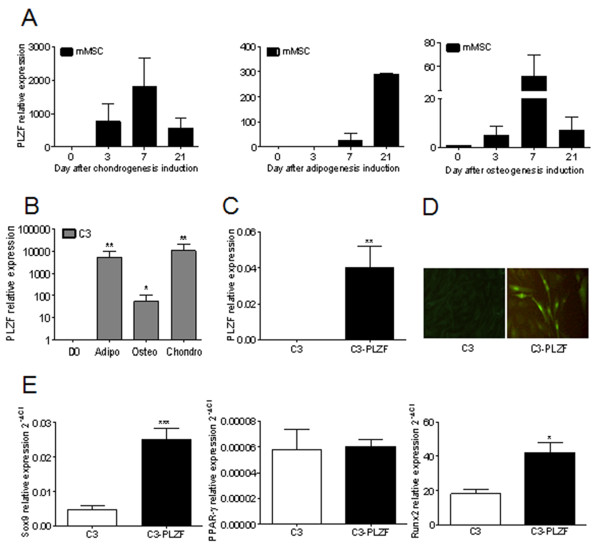
**Expression level of PLZF in primary mMSC, C3, and C3-PLZF cells. (A)** PLZF expression in mMSCs during chondrogenic, adipogenic, and osteogenic differentiation as assessed by RT-qPCR. **(B)** Expression profile of PLZF in undifferentiated (D0) and differentiated C3 cells into three lineages; adipogenic (Adipo), osteogenic (Osteo), and chondrogenic cells (Chondro). **(C)** Expression level of PLZF in undifferentiated C3 cells and in a clone of C3 cells with high PLZF expression (C3-PLZF), as quantified by using RT-qPCR. **(D)** Immunofluorescence analysis of C3 and C3-PLZF by using an anti-PLZF antibody. **(E)** Expression profile of the master regulators of the chondrogenic, adipogenic, or osteogenic programs, Sox9, PPARγ, or Runx2, respectively, in the C3 or C3-PLZF cells.

The increased expression of PLZF during MSC commitment suggested that it might play a major role in the differentiation processes. To address this issue, we tested whether PLZF overexpression may enhance the differentiation of MSCs toward chondrocytes, osteoblasts, or adipocytes. For that purpose, we used murine BM-derived MSCs (mMSCs) and investigated the expression profile of PLZF during their differentiation process toward the three lineages. We showed that PLZF expression reached its maximum at day 7 of osteogenesis and chondrogenesis to slightly decrease at day 21 (Figure 3A). During adipogenesis, PLZF expression was induced at day 7 to increase further until the end of the differentiation (Figure 3A).

In parallel, by using the well-characterized mouse MSC line, C3H10T1/2 (here called C3 cell), we also demonstrated that PLZF was not expressed in the undifferentiated cells but was significantly upregulated in the cells induced to differentiate into the three lineages (Figure 3B). We therefore decided to use C3 cells to study the role of PLZF in MSC differentiation. First, we transfected C3 cells to obtain MSC that stably overexpress PLZF. We selected several clones that were resistant to hygromycin B and expressed high levels of PLZF. We identified a high-expressing clone, called C3-PLZF, that was thereafter used in the study (Figure 3C, D). We then investigated whether PLZF overexpression could influence the expression level of the master regulators of the adipogenic, osteogenic, or chondrogenic programs. We showed that Sox9 and Runx2, key transcription factors of chondrogenesis and osteogenesis, respectively, were significantly increased in C3-PLZF compared with C3 cells, whereas the master regulator of adipogenesis, PPAR-γ, was unchanged (Figure 3E). Altogether, these data suggest that PLZF acts as a transcription factor regulating both Sox9 and Runx2 TF, suggesting that it might be a marker of chondro-osteoprogenitor cells.

### PLZF regulates the differentiation potential of MSCs

**Figure 4:**
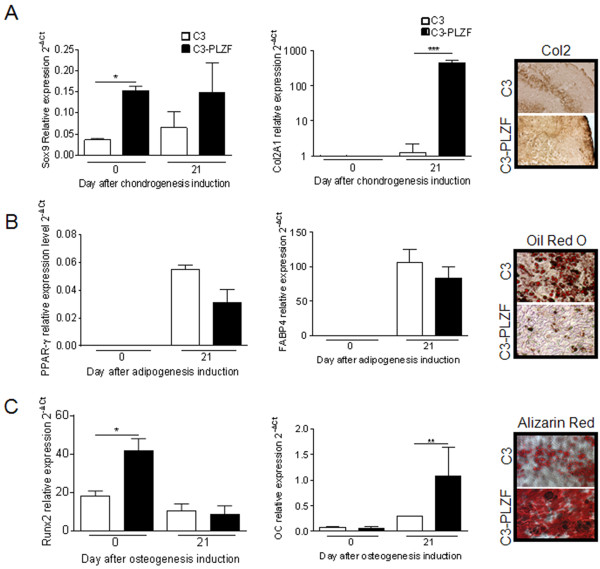
**Role of PLZF on MSC differentiation. (A)** Effect of PLZF overexpression on MSC chondrogenic differentiation after 21 days in micropellet culture. Expression of Sox9 and the cartilage marker Col2A1 in C3 and C3-PLZF, cells assessed by RT-qPCR and immunohistochemical analysis for collagen II expression in pellets. **(B)** Effect of PLZF overexpression on MSC adipogenic potential. Expression level of PPAR-γ and the adipocyte marker (FABP4) determined by RT-qPCR and staining of lipid droplets with oil red O. **(C)** Effect of PLZF overexpression on MSC osteogenic potential. Expression level of Runx2 and the osteoblast marker osteocalcin (OC), determined by RT-qPCR, and alizarin red S staining for mineralization. RT-qPCR data represent the mean ± SEM of three independent experiments.

We then determined whether PLZF overexpression could modulate MSC differentiation potential. We thus compared the capacities of C3 and C3-PLZF to differentiate toward the chondrogenic, osteogenic, and adipogenic lineages. C3 and C3-PLZF cells induced to differentiate into chondrocytes, formed a similar pellet, with an increased production of type II collagen (Col2) (Figure 4A). Staining in C3-PLZF-derived pellets was uniform throughout the pellet, whereas it was localized in the center of the pellets obtained with C3 cells, suggesting a more homogeneous differentiation of C3-PLZF cells. This observation was supported by the significant increase in Col2A1 mRNA expression in pellets formed with C3-PLZF cells, as compared with C3 (Figure 4A). MSCs cultured in adipogenic medium formed characteristic intracellular lipid-rich vacuoles that stained positive with oil red O. Compared with C3, C3-PLZF displayed a substantially lower ability to form lipid vacuoles suggesting a reduced degree of adipocytic maturation (Figure 4B). However, we showed that C3-PLZF differentiated into adipocytes expressed a similar expression level of fatty-acid-binding protein (FABP)-4 and PPAR-γ (Figure 4B). Finally, C3-PLZF induced to undergo osteogenesis expressed a higher mRNA expression level of osteocalcin (OC) at day 21 than did C3 cells (Figure 4C). Moreover, under osteogenic conditions, cells cultured for 21 days produced a mineralized matrix that stained positive with alizarin red, with the highest level of mineralization seen in C3-PLZF cultures (Figure 4C). In summary, PLZF overexpression in C3 resulted in enhanced chondrogenesis and osteogenesis, whereas it inhibited adipocyte maturation.

### PLZF-overexpressing MSCs formed cartilage and bone *in vivo*

**Figure 5:**
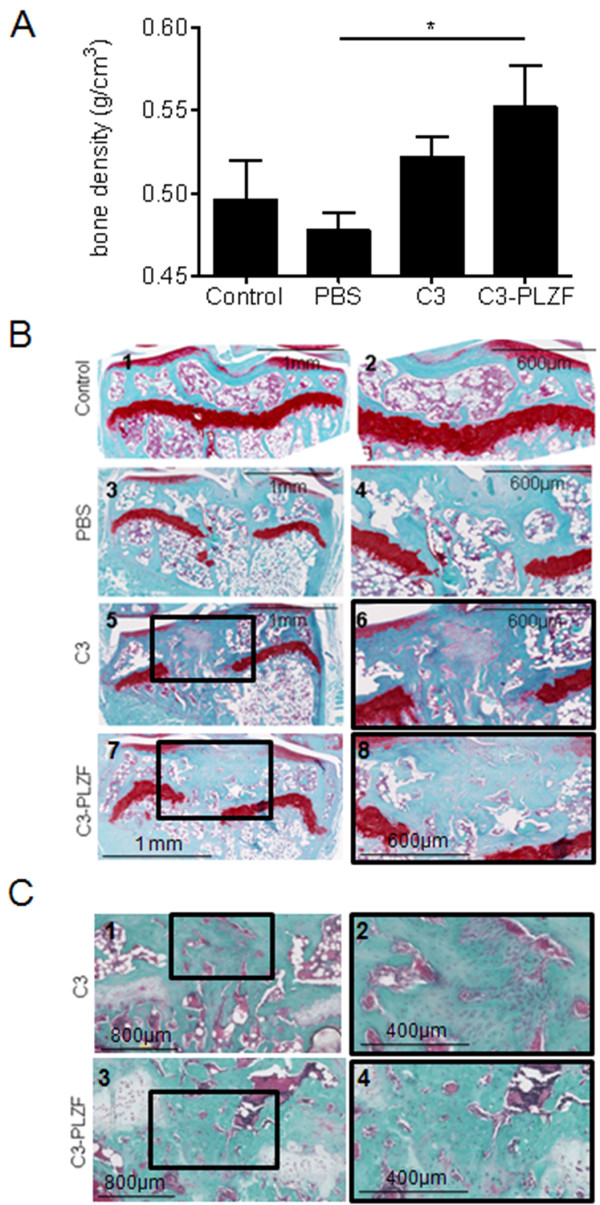
**C3-PLZF cell injection leads to bone formation in an osteoarticular defect model in immunocompetent mice. (A)** Quantification of the bone density (g/cm^3^). **(B)** Histologic analysis of tibial sections stained with Safranin O and Fast green. On the top, a representative control tibia. **(C)** Goldner trichrome histologic staining. Tibias were recovered 28 days later.

Finally, we determined whether PLZF-overexpressing C3 cells could promote the repair of osteochondral defects *in vivo*. We compared the effect of C3 and C3-PLZF injection in lesions that penetrated both the cartilage and bone through the epiphyseal and growth plates. At 28 days after cell implantation, hindlimbs of DBA/1 mice were scanned with a micro-computed tomograph to evaluate bone mineral density (BMD) before histologic evaluation. A significant increase of the BMD was observed in mice receiving C3-PLZF compared with the group of mice injected with PBS (Figure 5A). Safranin O (Saf O) staining of sections revealed a substantial replenishment of the osteochondral defect with trabecular bone and bone marrow in the group receiving PBS (Figure 5B3 and B4). After C3 cell injection, a mix of fibrous tissue and bone filled with bone marrow was seen by using Saf O staining (Figure 5B5 and B6) and Goldner trichrome staining for bone identification (Figure 5C1 and C2). In contrast, in the group of mice treated with C3-PLZF cells, the defect was filled mostly by bone tissue with a few cavities of bone marrow as seen by Saf O (Figure 5B7 and B8) and Goldner trichrome staining (Figure 5C3 and C4).

**Figure 6:**
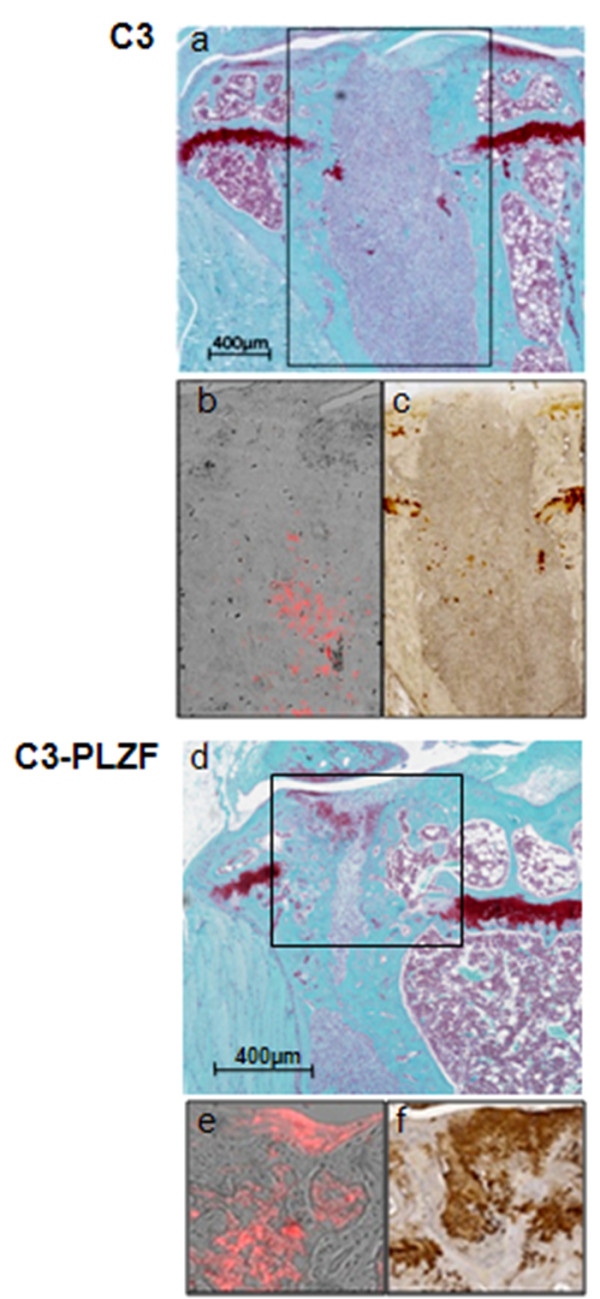
**C3-PLZF cell injection leads to cartilage and bone formation in an osteoarticular defect model in SCID mice. (a)** A representative histologic section of the tibia of a SCID mouse that has received naïve C3 cells stained with Safranin O and Fast green. **(b)** Distribution of CM-DiI-labeled C3 shown by fluorescence microscopy as red fluorescent cells. **(c)** Collagen II-positive staining on tibia sections. **(d)** Histologic sections of tibias stained with Safranin O and Fast green. C3-PLZF cell injection leads to cartilage formation in an osteoarticular defect model in SCID mouse. **(e)** Distribution of CM-DiI-labeled C3-PLZF shown by fluorescence microscopy as red fluorescent cells. **(f)** Collagen II-positive staining on tibia sections.

A similar experiment was also conducted in SCID/Bg mice. To determine the origin of the neotissue, we stained the cells with the fluorescent marker CM-DiI before injection. We showed in this model that C3 injection led to the formation of fibrous tissue with some fluorescent C3 cells in the neotissue. No formation of cartilage extracellular matrix was observed, as revealed by the absence of proteoglycans after Saf O or Col2 staining (Figure 6a and c). When C3-PLZF cells were injected, the defects were filled with cartilage-like tissue that stained positive for Saf O on the superficial layer (Figure 6d). At higher magnification, we observed the presence of newly differentiated chondrocytes producing Col2-positive matrix and some fibrous tissue (Figure 6f). Moreover, presence of CM-DiI-positive injected cells was observed in the neoformed tissue (Figure 6b and e). Indeed, we showed that a number of the cells that differentiated into chondrocytes were the injected C3 or C3-PLZF, as observed by the red fluorescence. However, the fluorescent staining of cells did not cover all the surface of the newly formed tissue. Therefore, whereas C3-PLZF injection resulted in the formation of bone in immunocompetent mice, it gave rise to cartilage-like tissue in SCID mice.

## Discussion

We demonstrated the link between PLZF expression and MSC commitment during their differentiation toward the three main lineages. Indeed, we showed that although the absence of expression of PLZF in MSCs signed their undifferentiated state, its early induction predicted their commitment toward adipogenesis, chondrogenesis, and osteogenesis. Moreover, this study provided insights into the molecular mechanisms underlying MSC fate determination. We showed that PLZF acted upstream of Sox9 and Runx2, and *in vitro*, its overexpression in MSCs increased both their chondrogenic and osteogenic potential while inhibiting adipogenesis. *In vivo*, implantation of PLZF-overexpressing MSCs into an intratibial osteochondral defect formed bone and cartilage. Our findings have therefore important implications for the role of PLZF in MSC biology and in particular in their chondrogenic and osteogenic differentiation potential.

MSCs are an attractive source for cell therapy and tissue engineering. However, currently, no marker specific for MSCs has been identified. Herein, we demonstrated that MSCs as well as, hESCs and iPSs, share in common the absence of PLZF expression. We further showed a strong relation between PLZF expression and lineage commitment. Early upregulation of PLZF after induction of differentiation of MSC toward the three lineages was observed independently of the cell source, except during osteogenic differentiation of ASC.

Another difference between cell sources was noticed for the expression profile of PLZF during chondrogenesis of BM-MSC. The reason for these disparities is not known, but we hypothesized that PLZF may exert different roles, depending on the source of cells. Indeed, *in vitro*, by using both human and mouse MSCs we showed an induction of PLZF as early as day 1 of the chondrogenic, osteogenic, and adipogenic differentiation processes. This result suggests that PLZF might be used to interrogate molecularly the early stages of MSC commitment. Moreover, we showed that PLZF overexpression affected MSC differentiation potential because it significantly increased chondrogenesis and osteogenesis, whereas it inhibited the maturation of adipocytes. These results are in line with the fact that PLZF overexpression in C3 cells is associated with a significant increase in the level of Sox9 and Runx2, but it did not change the PPAR-γ expression profile, as well as previous studies highlighting the role of PLZF in cartilage [10] or bone formation [12]. MSC differentiation is controlled by the regulated activity of transcription factor networks, among which, Sox9 and Runx2 cooperate in a tightly and temporally regulated manner. The interesting finding of the present study is the possibility that PLZF may act upstream of these specific transcription factors. It is likely that such a role is not through direct binding of PLZF to regulatory sequences in the promoter region of Sox9 or Runx2 but via interactions with other factors. We therefore propose that PLZF is an early marker of MSC commitment, which acts as an upstream regulator of Sox9 and Runx2, promoting chondrogenesis and osteogenesis, respectively.

Interestingly *in vivo*, we showed that the intratibial implantation of C3-PLZF cells was accompanied by excessive bone formation in immunocompetent mice, whereas it filled the defects with a reparative cartilage-like tissue in the superficial layer in immunodeficient mice. The newly formed tissue was partly associated with the presence of the injected cells that were most numerous after injection in immunodeficient mice. We cannot determine whether the injected MSCs stimulated the recruitment and differentiation of endogenous cells to promote bone or cartilage formation or whether they proliferated *in situ* and subsequently lose the fluorescent staining. Interestingly, the nature of the neotissue formed after C3-PLZF injection depended on the host immune status.

In the present study, we used the SCID/bg mouse strain, which lacks mature T cells, B cells, natural killer cell (NK) activity, and might harbor macrophage defects [31,32]. Physiologically, mature osteoblasts arising from MSCs after the activation of different transcription factors, such as Runx2, can regulate osteoclast activity. Both cell types are involved in bone remodeling, which is further regulated by the immune system, and lymphocyte- or macrophage-derived cytokines are among the most potent mediators of osteoimmunologic regulation. Indeed, the bone-protective role of resting T lymphocytes has been demonstrated in T-cell-deficient mice, which displayed a significant increase in basal osteoclast number and reduced bone density as compared with controls [33]. Furthermore, *in vivo* depletion of CD4^+^ and CD8^+^ T cells enhances osteoclast formation by a mechanism involving suppression of osteoprotegerin production by B cells [34]. Among the most prominent cytokines produced by NK cells are tumor necrosis factor-α (TNF-α) and interferon γ (IFN-γ), adding to the fact that the SCID/bg mouse environment is reduced in inflammatory stimuli [35,36]. Cytokines produced by activated monocytes/macrophages have been described to induce osteoblast differentiation and matrix mineralization from MSCs [37]. This observation is in line with studies revealing that osteal macrophages involved in bone formation or healing are inflammatory and produce high amounts of TNF-α [38,39]. Irrespectively, the cellular mediators from macrophages were also shown to act in an autocrine and paracrine fashion to induce imbalance between bone formation and resorption, either by enhancing the osteoclastic lineage or by acting on stromal or osteoblastic cells, leading to the loss of bone stock [40,41]. Moreover, IL-1β and TNF-α display potent NF-κB–dependent inhibitory effects on cartilage formation [42]. These different studies highlighted the fact that immune cells are necessary for bone homeostasis. Similar findings were observed in the present study, in which osteoblastogenic differentiation of MSCs took place after intratibial injection in immunocompetent mice, likely via the secretion of bone-inducing factors secreted by the surrounding environment. In this model, bone formation was enhanced when PLZF overexpressing MSCs were implanted. On the contrary, in immunodeficient mice, MSC differentiation was inhibited or greatly reduced, and PLZF overexpression favored cartilage formation instead of bone formation. The present results are in agreement with the findings made by Liu and coworkers on the role of PLZF as a factor increasing cartilage repair in an osteochondral defect model [10].

Our study asked furthermore about the contribution of immune cells to the role of PLZF in MSC differentiation. In addition to the role of immune cells on the balance between bone formation and bone resorption, which has been well documented, it may be possible that absence of T cells may shift the differentiation program of MSCs toward cartilage instead of bone formation. This hypothesis, however, needs further investigation.

## Conclusion

This study demonstrated that PLZF expression is lacking in undifferentiated stem cells and induced early during the differentiation of MSCs toward the three main lineages. We showed that PLZF induction in MSC acted as a molecular switch between osteo-chondroprogenitor and adipogenic progenitor cell fates, according to environmental conditions. Hence, PLZF could be a key regulator for the “stemness” maintenance of stem cells and act as an inducer of MSC commitment. This finding improves our knowledge on the dual role of PLZF in MSC differentiation potential and paves the way for developing specific therapeutic approaches for cartilage and bone repair.

## Abbreviations

ASC: Adipose tissue-derived MSC; BMD: bone mineral density; BMP: bone morphogenetic protein; Col2: type II collagen; DMEM: Dulbecco modified eagle medium; ESC: embryonic stem cell; FABP: fatty-acid-binding protein; FCS: fetal calf serum; FGF: fibroblast growth factor; iPS: induced pluripotent stem cell; MEM: minimum essential medium; MSC: mesenchymal stem cell; OC: osteocalcin; PBS: phosphate-buffered saline; PCR: polymerase chain reaction; PLZF: promyelocytic leukemia zinc-finger; PPAR: peroxisome proliferator-activated receptor; RPS9: ribosomal protein S9; Saf O: safranin O; TF: transcription factor; TGF: transforming growth factor.

## Competing interests

The authors declare no competing financial interests.

## Authors’ contributions

FD participated in the performance of all experiments, interpreted and analyzed the results, and wrote the manuscript; GT, MM, and CB performed the chondrogenic, osteogenic, and adipogenic experiments by using human and mouse MSCs and participated in data analysis. KT participated in the experiments on the mouse osteochondral defect model, evaluated the bone mineral density (BMD) with the microcomputed tomography, and participated in data analysis. AB performed the Goldner trichrome staining for bone identification. PC performed the microarray analysis. CJ participated in the conception, the design of the experiments, and critically revised the content of the manuscript. DN designed the research, interpreted the data, and wrote the manuscript. All authors read and approved the final manuscript.
